# Horizontal Transfer of Small RNAs to and from Plants

**DOI:** 10.3389/fpls.2015.01113

**Published:** 2015-12-10

**Authors:** Lu Han, Yu-Shi Luan

**Affiliations:** School of Life Science and Biotechnology, Dalian University of TechnologyDalian, China

**Keywords:** miRNA, siRNA, horizontal gene transfer, plant-microbe interactions, controversy of cross-kingdom miRNA

## Abstract

Genetic information is traditionally thought to be transferred from parents to offspring. However, there is evidence indicating that gene transfer can also occur from microbes to higher species, such as plants, invertebrates, and vertebrates. This horizontal transfer can be carried out by small RNAs (sRNAs). sRNAs have been recently reported to move across kingdoms as mobile signals, spreading silencing information toward targeted genes. sRNAs, especially microRNAs (miRNAs) and small interfering RNAs (siRNAs), are non-coding molecules that control gene expression at the transcriptional or post-transcriptional level. Some sRNAs act in a cross-kingdom manner between animals and their parasites, but little is known about such sRNAs associated with plants. In this report, we provide a brief introduction to miRNAs that are transferred from plants to mammals/viruses and siRNAs that are transferred from microbes to plants. Both miRNAs and siRNAs can exert corresponding functions in the target organisms. Additionally, we provide information concerning a host-induced gene silencing system as a potential application that utilizes the transgenic trafficking of RNA molecules to silence the genes of interacting organisms. Moreover, we lay out the controversial views regarding cross-kingdom miRNAs and call for better methodology and experimental design to confirm this unique function of miRNAs.

## Introduction

The fundamental concept of gene transfer is that it occurs from parents to offspring. In addition to this vertical transfer, horizontal gene transfer has also been shown to exist in bacteria and simple eukaryotes (Gogarten et al., 2002; Anderson, 2009). Recently, studies have indicated that genes from viruses, prokaryotes and fungi can be transferred to higher species, a phenomenon that has received a great attention (Yue et al., 2012; Crisp et al., 2015). As the products of gene transcription, small RNAs (sRNAs) have been reported to move horizontally between different species. sRNAs of approximately 19–25 nucleotides in length belong mainly to two classes: microRNAs (miRNAs) and siRNAs. Acting as regulatory molecules, sRNAs are involved in a wide range of biological processes that are essential for organ morphogenesis, genome modification, and adaptive responses to biotic and abiotic stresses (Reinhart et al., 2002; Carrington and Ambros, 2003; Lai, 2003; Bartel, 2004). In plants and animals, sRNAs direct the cleavage of endogenous mRNAs or repress their translation (Hamilton et al., 2002; Llave et al., 2002). In addition, sRNAs protect plants and animals from viral infections through the RNA interference (RNAi) system (Wang et al., 2004). It is believed that RNAi also functions in communication among different kingdoms. Recently, both animals and plants have been reported to exchange sRNAs with parasites, pathogens, or symbiotic organisms. Many studies have reported the introduction of cross-kingdom sRNAs between animals and parasites. For example, miRNAs traffic from human sickle cells to malarial parasites (LaMonte et al., 2012) and from helminth nematodes to mouse cells (Buck et al., 2014). However, compared with animals, trafficking of sRNAs has not been widely observed between plants and other organisms. Additionally, plant sRNAs are mobilized through the phloem and are carried to distinct target cells, where the sRNAs induce a reduction of gene expression (Molnar et al., 2010). Therefore, it is of great value to investigate the mobility of sRNAs between different species.

According to computational analysis and experimental validation, certain types of plant-associated sRNAs have been shown to cross kingdoms and play a role in improving immunity and the defense against viruses or the aggravation of viral symptoms. In this report, we describe the processes and effects of plant-derived miRNAs that move to animals/viruses and microbe-derived siRNAs that move to plants, including host-induced gene silencing (HIGS) system that utilize sRNAs to silence parasite genes in plants. Moreover, we discuss the competing view of cross-kingdom miRNAs and introduce the main techniques employed to measure exogenous miRNAs, and call for better methodology and experimental design to confirm this unique function of miRNAs.

## Roles of Horizontally Transferred miRNAs

### miRNAs Transferred from Plants to Animals

**FIGURE 1:**
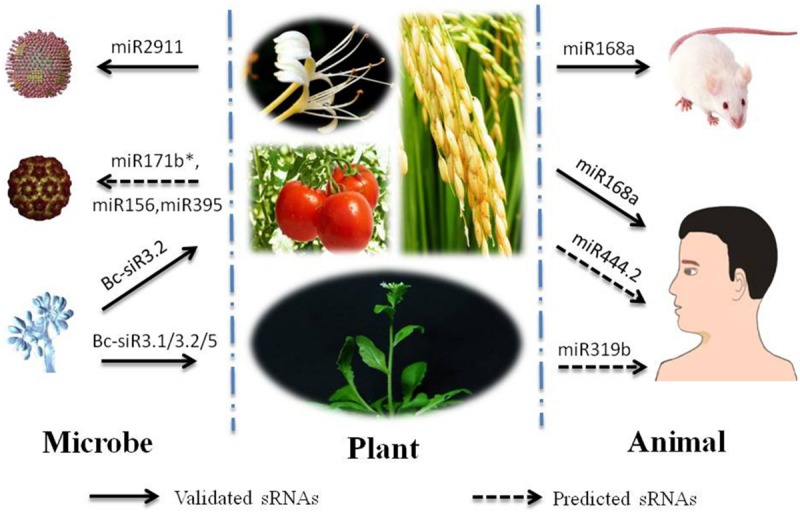
**Trafficking of typical cross-kingdom small RNAs (sRNAs) involved with plants.** Nine organisms are selected for depiction of cross-kingdom sRNAs involved with plants. Among them, three organisms of ‘Microbe’ column top-down are *Influenza A viruses* (IAV), CMV and *Botrytis cinerea.* Four organisms of ‘Plant’ column are *Loniceraja ponica (L. ponica), Solanum lycopersicum*, and *Oryza sativa* on the first line and *Arabidopsis thaliana* on the second line. Two organisms of ‘Animal’ column are *Mus musculus (M. musculus)* and *Homo sapiens(H. sapiens).* Cross-kingdom sRNAs are listed in **Table 1**. The arrows under the cross-kingdom sRNAs point to target species.

Dietetically absorbed plant miRNAs have been confirmed to exist stably in human plasma (Liang et al., 2014a). Thus, it is an intriguing question whether these evolutionarily conserved plant miRNAs can enter into mammalian cells and exert physiological functions. Indeed, it has been demonstrated by high-throughput sequencing that certain miRNAs from plants such as *Zea mays (Z. mays), Arabidopsis thaliana (A. thaliana), Oryza sativa (O. sativa)*, and *Citrus trifoliate (C. trifoliate)* can exist stably in human plasma and breast milk exosomes, including 35 miRNAs from 25 MIR families (Lukasik and Zielenkiewicz, 2014). Targets of the aforementioned miRNAs included the mRNAs of proteins associated with transcription factors (e.g., low-density lipoprotein receptor, LDLR), immune system functions (e.g., zinc finger e-box-binding homeobox 1, ZEB1), saccharometabolism (e.g., glycogen debranching enzyme, GDE) and hormone responses (e.g., melanocortin receptor 4, MC4R; **Table 1**, **Figure 1**). Due to the vital role of breast milk in infant growth and nutrition, it is of great value to explore the effects of foreign miRNAs on infants. It has been proven that plant miRNAs are consistently present in the umbilical cord blood and amniotic fluid of humans (Li et al., 2015). This suggests that those plant miRNAs may transfer through the placenta to the fetus. Moreover, a greater number of immune-related miRNAs have been detected in the colostrum than in mature milk (Gu et al., 2012). Therefore, certain immune-related exogenous miRNAs that may exist in the colostrum are expected to influence an infant’s immune system.

**Table 1:** Trans-kingdom small RNAs.

Number/Name of sRNAs	Derivation	Target species	Target genes/ORFs	Reference
35 miRNAs	*Z. mays, A. thaliana*,	*H. sapiens*	LDLR, ZEB1, GDE,	Lukasik and Zielenkiewicz, 2014
(e.g., zma-miR156a,	*O. sativa, C. trifoliate*,		MC4R, etc.	
ath-miR319b, osa-miR444.2,	etc.			
ctr-miR167)				
38 miRNA/miRNA^∗^	*S. lycopersicum*	CMV-Fny	2a, 3a ORFs	Feng and Chen, 2013
(e.g., miR171b^∗^, miR156)				
37 miRNA/miRNA^∗^	*S. lycopersicum*	CMV-Q	2a, 3a ORFs	Feng and Chen, 2013
(e.g., miR171b^∗^, miR395)				
8 miRNA/miRNA^∗^	*S. lycopersicum*	ToLCNDV	AC1, AC2, AC3,	Naqvi et al., 2010
(e.g., miR1918, miR156b^∗^, miR164a, miR172b^∗^, miR166a^∗^)			AV1, AV2, BV1 ORFs	
miR168a	*O. sativa*	*H. sapiens*/*M. musculus*	LDLRAP1	Zhang et al., 2012a
miR2911	*L. ponica*	IAVs	*PB2* and *NS1*	Zhou et al., 2014
vsiR1378	GFkV	*V. vinifers*	S2P metalloprotease	Miozzi et al., 2013
vsiR6978	GRSPaV	*V. vinifers*	VPS55	Miozzi et al., 2013
siR221	TMV-Cg	*A. thaliana*	CPSE30	Qi et al., 2009
siR118	TMV-Cg	*A. thaliana*	TRAPα	Qi et al., 2009
vsRNA (termed as sRCC1)	CaMV	*A. thaliana*	*At1g76950* (awaits functional characterization)	Moissiard and Voinnet, 2006
Bc-siR3.2	*B. cinerea*	*A. thaliana*	MPK2 and MPK1	Weiberg et al., 2013
Bc-siR3.1	*B. cinerea*	*A. thaliana*	PRXIF	Weiberg et al., 2013
Bc-siR5	*B. cinerea*	*A. thaliana*	WAK	Weiberg et al., 2013
Bc-siR3.2	*B. cinerea*	*S. lycopersicum*.	MAPKKK4	Weiberg et al., 2013
Y-sat derived siRNA	CMV	*N. tabacum*	*ChlI*	Shimura et al., 2011; Smith et al., 2011

*Predicted sRNAs of shadow area and validated sRNAs are listed in the first column. The direction of trans-kingdom sRNAs above is from ‘Derivation’ to ‘Target Species.’ Some predicted sRNAs are of large quantities and we do not spread them in the table, just give the number and representative ones, each corresponding to their target genes/ORFs, except of the underlined miR156b^∗^ targeting two ORFs, AC2, and AC3.*

In another study, Zhang et al. (2012a) demonstrated that single-stranded mature miRNAs present in rice can exist stably in the sera and tissues of various animals and humans. One of these miRNAs, osa-miR168a, has been shown to be selectively packed into microvesicles (MVs; Liang et al., 2010) that were shed from intestinal epithelial cells and then released into the circulatory system. These MVs efficiently delivered the miRNAs into recipient cells, in which osa-miR168a suppressed the expression of a target gene, low-density lipoprotein receptor adapter protein 1 (LDLRAP1), in the livers of humans and mice, thereby decreasing the removal of low-density lipoprotein from the plasma (**Table 1**, **Figure 1**). In addition to miR168a, another type of plant miRNA, miR172, has been observed in the stomach, intestine, serum, and feces of mice after being fed total RNA extracted from *Brassica oleracea* (Liang et al., 2014b), which suggests that plant miRNAs can survive in the circulatory system and gastrointestinal (GI) tract in mice. Interestingly, synthetic tumor suppressor miRNAs that mimic plant miRNAs can be absorbed by GI tract and functions in reducing tumor burden of mice (Mlotshwa et al., 2015). This method could be utilized to produce edible plants that contain therapeutic tumor miRNAs, which may be applied as clinical small molecules for patient treatment.

### miRNAs Transferred from Plants to Viruses

In addition to plant miRNAs that can transfer into the bodies of humans and mice, there are other types of miRNAs that have been speculated to move in a trans-kingdom manner from plants to viruses. Feng and Chen (2013) predicted a total of 38 and 37 tomato miRNA/miRNA^∗^ sequences that mostly shared high complementarity with the open reading frames (ORFs) of the genomic RNAs of CMV-Fny (severe subgroup 1A strain) and CMV-Q (mild CMV subgroup strain), respectively, which may result in repression of translation or the cleavage of target genes. Importantly, some of these genes act in CMV replication (e.g., CMV protein 2a) and movement (e.g., CMV protein 3a; **Table 1**, **Figure 1**). It has been assumed that plants utilize the mechanism of post-transcriptional gene silencing (PTGS) to prevent the replication and spread of CMV virions. Similar results have been obtained in *Tomato leaf curl New Delhi virus* (ToLCNDV), in which six encoded ORFs of DNA-A and DNA-B were shown to be targeted by eight miRNA/miRNA^∗^ sequences (Naqvi et al., 2010; **Table 1**).

Computational prediction alone cannot demonstrate that certain plant miRNAs crossover to viruses. Plant miR2911, the only miRNA that exists stably in honeysuckle decoction (HS decoction) due to its special high G-C content, has been shown to target the genes of *Influenza A viruses* (IAVs) with the help of MVs in humans and mice (Zhou et al., 2014). Drinking HS decoction results in a significant increase in miR2911 levels in the plasma and lungs of mice. Plant miR2911 can directly bind to the target genes *PB2* and *NS1*, which are essential for influenza replication, thereby inhibiting their amplification (**Table 1**, **Figure 1**). Notably, the transport of miR2911 has been shown to be similar to that of miR168a, as both are packed into MVs and go through the GI tract. Afterwards, these miRNAs are transported to target cells through the circulatory system.

### The Probable Process of the Horizontal Transfer of miRNAs from Plants to Mammals

When we ingest plant materials, they are preliminarily crushed into debris by the mechanical action of the oral cavity and stomach and simultaneously catabolized into glucose by various digestive enzymes. It has been assumed that, in this process, mature miRNAs are released from the destroyed plant cells and transferred to the small intestine in the gut (Zhang et al., 2012a). Along with AGO2, these miRNAs have been observed to selectively pack into shedding vesicles or exosomes (both called MVs) and are secreted to the outer space by epithelial cells (Cocucci et al., 2009; Elhassan et al., 2012; Zhang et al., 2012a; Zhou et al., 2014). It is worth noting that not all of the miRNA-AGO2 complexes are packed into MVs when entering into the intestinal epithelial cells, and only a minority of the complexes in the MVs can gain access to the recipient cells (Collino et al., 2010). Indeed, exosomal miRNAs are derived from a particular subset of genes (Valadi et al., 2007). In some cases, the packaging of miRNAs into MVs is driven by particular substances, such as an antigen (Mittelbrunn et al., 2011). Via endocytosis and exocytosis, MVs translocate through the vascular wall and are transported to the target cells via the circulatory system, with the ensuing step of releasing the miRNA-AGO2 complexes. Nevertheless, MVs do not interact with all types of cells and interact only with those cells that the MVs specifically recognize. Because foreign miRNAs have high G-C content and specific 2′-*O*-methylated 3′ ends (Yu et al., 2005), and their transportation is with the help of binding to AGO2 and shielding in MVs (Mitchell et al., 2008; Arroyo et al., 2011; Zhang et al., 2012a; Zhou et al., 2014), they can exist stably under different temperatures, acidification and RNase activity during the process of digestion and transportation.

## Roles of Horizontally Transferred siRNAs

### siRNAs Transferred from Viruses/Fungi to Plants

Virus-derived siRNAs bind to plant transcripts. vsiRNAs from *Grapevine fleck virus* (GFkV) and *Grapevine rupestris stem pitting-associated virus* (GRSPaV) have been predicted to target plant transcripts according to genome-wide identification (Miozzi et al., 2013). It has been reported that 24/26 different grapevine transcripts could be targeted by 27/30 vsiRNAs from the GFkV genome and GRSPaV genome, respectively. To test whether the decrease in grapevine transcripts described above was related to the infection of viruses, Miozzi et al. (2013) carried out quantitative real-time polymerase chain reaction (qRT-PCR) and 5′-RACE analyses. The results supported the idea that a lower accumulation of transcripts, such as S2P metalloprotease and vacuolar protein-sorting 55 (VPS55), was associated with the cleavage of vsiRNA from GFkV (vsiR1378) and GRSPaV (vsiR6978) (**Table 1**). The former transcript is implicated in the control of regulated intramembrane proteolysis, and the latter is involved in the regulation of the endosomal trafficking of proteins. In the same manner, *Tobacco Mosaic Virus* (TWV-Cg) siR221 and siR118 target cleavage and polyadenylation specificity factor (CPSE30) and translocon-associated protein alpha (TRAPα) in *A. thaliana*, respectively (Qi et al., 2009) (**Table 1**). Two targets of the siRNAs were validated through modified RNA ligase-mediated 5′-RACE experiments. In addition to the RNA viruses described above, sRNAs from the DNA virus *Cauliflower Mosaic Virus* (CaMV) can also silence plant transcripts (Moissiard and Voinnet, 2006). The CaMV-derived sRNAs mostly come from the polycistronic 35S RNA sequence, which exhibits an extensive secondary structure known as a translational leader. By employing the entire leader sequence in a BLAST search against cDNAs and ESTs from *Arabidopsis*, three transcripts (*At4g05190, At4g17710, and Atlg76950*) were retrieved. One of these transcripts, *Atlg76950*, was confirmed to bind the corresponding vsiRNA (termed as sRCC1; **Table 1**).

Fungus-derived siRNAs bind to plant transcripts as well. *Botrytis cinerea* (*B. cinera*) sRNAs (Bc-sRNAs) that silence plant genes involved in immunity are an example. Normally, pathogens deliver protein effectors into plant cells to suppress plant immunity. However, sRNAs derived from *B. cinera* may also act as effectors (Weiberg et al., 2013). In *A. thaliana*, three Bc-sRNAs (Bc-siR3.1, Bc-siR3.2, and Bc-siR5) that structurally mimic plant sRNAs can be loaded into the plant AGO1 protein, after which the Bc-sRNAs target genes with complementary sequences, such as *mitogen-activated protein kinase 2* (MPK2) and MPK1 (by Bc-siR3.2); an oxidative stress-related gene, *peroxiredoxin* (PRXIIF; by Bc-siR3.1); and *cell wall-associated kinase* (WAK; by Bc-siR5), which are involved in the plant’s immunity against *B. cinera*. Similar results have been obtained in *Solanum lycopersicum*, where MAPKKK4 was targeted by Bc-siR3.2 (**Table 1**, **Figure 1**).

### siRNAs from CMV Satellite RNAs Transferred to Plants

Satellite RNAs (satRNAs), a type of subviral RNA, are encapsulated by their helper viruses, such as CMV. SatRNAs are dispensable for the replication of the genome/subgenome of viruses but have the ability to aggravate or attenuate disease symptoms (Simon et al., 2004). CMV exhibits a tripartite genome whose components are termed RNA1, RNA2, and RNA3, which are mainly required for its replication, virulence, and movement (Kouakou et al., 2013). CMV also contains subgenomic RNAs (RNA4 and RNA4A), which are each responsible for the translation of the coat protein and protein 2b. In addition to genomic and subgenomic RNAs, CMV strains encompass satRNAs, and one type of satRNA, Y satRNAs (Y-sat), can produce siRNAs that may be associated with yellowing symptoms caused by an RNAi mechanism in *Nicotiana tabacum (N. tabacum)*. Because subviral RNA has no ability to encode proteins, it is reasonable to postulate that RNA silencing mediates satellite pathogenicity (Wang et al., 2004). Shimura et al. (2011) and Smith et al. (2011) demonstrated that CMV Y-sat-derived siRNAs could interfere with the mRNA of the host magnesium protoporphyrin chelatase subunit I (*ChlI*) gene, leading to inhibition of chlorophyll biosynthesis, thus causing the yellow phenotype (**Table 1**).

## Host-Induced Gene Silencing (Higs) Acts as a Tool in the Defense Against Biotic Stress

It has been noted that plant inverted-repeat transgenic constructs, usually with a sense-intron-antisense palindromic structure, can be employed to produce dsRNAs and siRNAs and further silence the transcripts of parasitic organisms. Hence, such RNAi constructs could be designed to test whether HIGS can affect the interaction between plants and parasitic organisms. Based on experimental data, dsRNAs and siRNAs derived from transgenic constructs in host cells would be transferred to fungi/nematodes to achieve silencing of their genes (Nowara et al., 2010; Ibrahim et al., 2011; Yin et al., 2011; Koch et al., 2013; Ghag et al., 2014; Vega-Arreguín et al., 2014). HIGS, which is as an effective transgenic tool, has been used extensively to protect plants from infection by parasitic organisms (Nowara et al., 2010; Nunes and Dean, 2012; Ghag et al., 2014). It is still unknown how these RNA molecules are transferred to the interacting organisms. Nowara et al. (2010) considered it likely that these molecules may travel to the fungi via an exosomal pathway. On the one hand, exosomes are accumulating at plant-fungus contact sites and vesicle fusion/budding have been observed at the haustoria complex, which is responsible for the transfer of nutrients to the fungi. On the other hand, plant multivesicular bodies have been shown to contain small RNAs as well as components of the silencing machinery (Valadi et al., 2007). However, how RNA molecules gain access to the bodies of nematodes and target specific genes remains unknown and requires further elucidation.

## Controversies Related to Horizontally Transferred miRNAs that Remain to be Verified

The crossover of miRNAs is not universal between plants and animals. For example, insignificant plant miRNA levels have been detected in the plasma of healthy athletes and mice fed with fruits/vegetables (Snow et al., 2013). In addition, some plant miR168 family members were nearly undetectable when organisms were fed monocot plants in which miR168 levels were relatively higher (Zhang et al., 2012b). Besides, conflicting studies identified low levels of the aforementioned osa-miR168a that did not result in an RNAi-mediated decrease of LDLRAP1 in mouse livers (Dickinson et al., 2013; Witwer et al., 2013). Due to the controversies described above, we mainly focus on the research of Zhang et al. (2012a), who support the cross-kingdom function of miRNAs, and the research of Dickinson et al. (2013), who dispute this function, to examine the causes of the controversy. The experimental methods both show that deep sequencing and qRT-PCR are the main tools for measuring exogenous miRNAs. However, Zhang’s group conducted a more detailed experiment. The sequencing results obtained by Dickson may present a bias in plants, as only a thousand reads per million raw reads of rice sRNAs were detected in rice-containing chow, which is inconsistent with previous high-throughput sequencing studies showing that the miRNA reads obtained from rice generally represent 10% of total reads (Zhu et al., 2008; Jeong et al., 2011; Yi et al., 2013). Thus, it is not surprising that plant miRNAs cannot be detected in mouse sera and livers. As plant miRNAs bear 2′-*O*-methylated 3′ ends, which can result in a decreased ligation efficiency (Munafo and Robb, 2010), the deep sequencing results reported by Dickson need to be further verified, such as oxidized deep sequencing used by Zhang’s group to test genuine plant miRNAs, with the exception of those based on the qRT-PCR technique (Chen et al., 2013; Dickinson et al., 2013).

Apart from the two major controversial studies, many other studies involving cross-kingdom miRNAs have been conducted using similar methods. Some authors have been unable to detect or have only detected very small amounts of plant-derived miRNAs in mammals (Snow et al., 2013; Witwer et al., 2013), while others, who hold different views, claim that plant miRNAs exist in silkworms but do not play roles in physiological progress (Ling et al., 2015). It is worth noting that new techniques, known as next generation sequencing (NGS) and digital droplet PCR (dPCR), has been applied to test exogenous miRNAs successfully (Wang et al., 2012; Ling et al., 2015). Thus, the existing techniques and experimental design need to be modified for searching more exogenous miRNAs. Regarding the functions of exogenous miRNAs, studies have shown that the concentration of miRNAs affects their ability to target corresponding genes (Mullokandov et al., 2012). Therefore, the concentration of miRNAs in the species of origin has been a major limitation in the detection of miRNAs in other kingdoms thus far. Moreover, Dietary MicroRNA Database (DMD) presents for researchers the types and functions of dietary derived miRNAs, which will be a great tool to explore more of dietary miRNAs in the future (Chiang et al., 2015).

## Conclusion

The mobility of sRNA molecules is the key to understanding how sRNA molecules function in regulatory roles between one kingdom and another. In this process, the transportation of sRNAs through the MV pathway is shared both from plants to mammalians or from plants to fungi. Trafficking sRNAs derive from various species, including plants, viruses, and fungi. In most cases, crossover by these RNAs occurs in host-parasites interactions. Unlike miRNAs transferred from plants to mammals, these interactions are not one-sided, but bidirectional. It should be noted that the movement of RNA molecules has been used as a transgenic tool to control plant disease, such as through HIGS. Therefore, the elucidation of cross-kingdom sRNA mechanisms between two interacting organisms is of great interest. The horizontal transfer of sRNAs extends our understanding of sRNAs. In humans, this unique feature may be utilized to control viruses, such as *Influenza A* viruses, and improve infant immunity when consuming colostrum. For plants, the horizontal transfer of sRNAs can be used to control diseases caused by parasitic organisms. Although the number of plant-associated trafficking sRNAs is fewer compared with those of animals, and controversial views associated with dietary-derived miRNAs await validation, we still remain confident that an increasing number of foreign sRNAs will be studied and utilized in the future.

## Conflict of Interest Statement

The authors declare that the research was conducted in the absence of any commercial or financial relationships that could be construed as a potential conflict of interest.
